# The Potential Hepatoprotective Effect of Paeoniae Radix Alba in Thioacetamide-Induced Acute Liver Injury in Rats

**DOI:** 10.1155/2022/7904845

**Published:** 2022-01-28

**Authors:** Mi-Rae Shin, Se Hui Lee, Seong-Soo Roh

**Affiliations:** Department of Herbology, College of Korean Medicine, Daegu Haany University, Daegu 42158, Republic of Korea

## Abstract

**Aim:**

Acute liver injury (ALI) can occur for various reasons by induced inflammation and apoptosis of liver cells including hepatocytes, Kupffer cells, and hepatic stellate cells. Thioacetamide (TAA), which is a classic hepatotoxin, causes oxidative stress, membrane damage, and accumulation of lipid droplets and subsequently provokes consecutive liver injury. In the current study, we tested whether Paeoniae Radix Alba (PR) could alleviate TAA-induced ALI.

**Methods:**

Thirty-five male rats were equally separated into five groups. The first group was the normal group, which received distilled water only. The remaining four groups received intraperitoneal TAA (200 mg/kg) for 3 days to induce ALI. The four groups were divided into the control group (no treatment), silymarin-treated, 100 mg/kg PR-treated, and 200 mg/kg PR-treated. The efficacy of PR against hepatotoxicity was evaluated in terms of the serum biochemical index and protein expression associated with inflammation and apoptosis. Moreover, the dissected livers were analyzed by hematoxylin and eosin stain.

**Results:**

PR alleviated liver dysfunction as evidenced by decreased levels of aspartate aminotransferase, alanine aminotransferase, and ammonia. Phosphorylated AMP-activated protein kinase (AMPK) and Sirtuin 1 (Sirt1) levels were obviously decreased in the TAA control group, whereas PR reversed these changes. PR also prevented deteriorative effects through inhibition of inflammation and apoptosis via nuclear transcription factor-kappa Bp65 (NF-*κ*Bp65) inactivation. Moreover, we found that the hepatoprotective effect of PR pretreatment was mediated by restoration of histopathological changes.

**Conclusion:**

PR efficiently blocked both the inflammatory response and apoptosis through activating the AMPK/Sirt1/NF-*κ*Bp65 pathway. Therefore, PR is considered a potential therapeutic agent against ALI.

## 1. Introduction

Acute liver injury (ALI) is characterized by a rapid loss of liver function without preexisting liver disease and is frequently life-threatening. A variety of causes of ALI have been identified, including infections, neoplasms, toxic factors, and drugs [1]. Emerging evidence suggests that oxidative stress (OS) is involved in the development of liver injury [2]. Thioacetamide (TAA) converts into a highly toxic reactive intermediate, thioacetamide S-dioxide, by cytochromes P450 (CYP450) enzymes. CYP450 yields OS and participates in drug-induced liver damage. Accordingly, thioacetamide S-dioxide toxicity will be partially blocked by CYP450 inhibitors [3]. Bi et al. [4] reported that Paeoniae Radix Alba (PR; the root of the plant *Paeonia lactiflora Pallas*, Bai Shao) reduced toxicity via the degradation of toxic alkaloids and enhanced efficacy associated with CYP450. PR has been used as a medicinal herb in traditional Chinese medicine and is also named “White Paeony.” Recently, PR has been used more widely than Radix Paeoniae Rubra (Red Paeony) [5]. The application of PR is widely reported to refresh blood circulation, regulate menstruation, alleviate pain, and nourish the liver [6, 7]. Moreover, recent studies demonstrated that the neuroprotective potential of PR against strychnine-induced neurotoxicity was related to the suppression of OS [8]. PR also effectively treated an experimental autoimmune hepatitis mouse model via inhibition of OS [9].

AMP-activated protein kinase (AMPK) is a major regulator of cellular energy balance. Once activated by an increase of the cellular AMP : ATP ratio [10], it suppresses the rate of anabolic ATP consumption and the synthesis of proteins and/or lipids and increases the rate of catabolic ATP production and the oxidation of fatty acids and/or mitochondrial biogenesis to preserve ATP levels for intracellular energy homeostasis [11]. AMPK is composed of heterotrimeric complexes such as catalytic *α*-subunits and regulatory *β*- and *γ*-subunits. Under metabolic stress, catalytic *α*-subunits are phosphorylated, and then gene expressions and enzyme activities are altered to initiate corrective responses [12]. Specifically, AMPK controls gene expression by modulating Sirtuins in coordination with another metabolic sensor, the NAD^+^-dependent deacetylase. Sirtuins control many biological processes, such as apoptosis, cellular senescence, endocrine signaling, glucose homeostasis, aging, longevity, and mitochondrial biogenesis [13]. In particular, Sirtuin 1 (Sirt1) regulates numerous activities including metabolism, mitochondrial function, inflammation, and apoptosis during OS [14]. Therefore, many scientists have been trying to find Sirt1 modulators that can activate Sirt1. In turn, decreased expression of Sirt1 exacerbates the loss of AMPK phosphorylation and triggers the overproduction of reactive oxygen species and inflammatory responses including NF-*κ*Bp65 transcription [15].

According to previous hepatoprotective studies using PR, Wang et al. [16] reported that it had a hepatoprotective effect by regulating the homeostasis of bile acids and homocysteine, and Lee et al. [17] reported that it exhibited a hepatoprotective effect through the inhibition of oxidative stress. However, the underlying mechanism of PR in rats with ALI has not been clearly established. Accordingly, we elucidated that the anti-inflammatory and antiapoptotic effects of PR via the regulation of the AMPK/Sirt1/NF-*κ*Bp65 pathway.

## 2. Materials and Methods

### 2.1. Materials

Silymarin, thioacetamide, and phenyl methyl sulfonyl fluoride were purchased from Sigma-Aldrich (St. Louis, MO, USA). The protease inhibitor solution and EDTA were purchased from Wako Pure Chemical Industries, Ltd. (Osaka, Japan). Sodium carbonate was purchased from Daejung Chemicals & Metals Co., Ltd. (Siheung, Korea). Sodium hydroxide was purchased from OCI Company Ltd. (Seoul, Korea). Phosphoric acid was purchased from Duksan Company (Ansan, Korea). The Pierce BCA Protein Assay kit was purchased from Thermo Fisher Scientific Inc. (Waltham, MA, USA). ECL Western Blotting Detection Reagents and pure nitrocellulose membranes were purchased from GE Healthcare (Chicago, IL, USA). The primary antibodies p-LKB1 (#3482), LKB1 (#3047), p-AMPK (#2531), AMPK (#2532), and Caspase-3 (#9662S) were purchased from Cell Signaling Technology (Danvers, MA, USA). The primary antibodies Sirt1 (SC-15404), NF-*κ*Bp65 (SC-372), Bax (SC-7480), Bcl2 (SC-7382), cytochrome C (SC-13156), survivin (SC-17779), MCP-1 (SC-28879), ICAM-1 (SC-1511-R), VCAM-1 (SC-13160), TNF-*α* (SC-1351), IL-6 (SC-57315), COX-2 (SC-19999), iNOS (SC-7271), histone (SC-8030), and *β*-actin (SC-4778) were purchased from Santa Cruz Biotechnology, Inc. (Dallas, TX, USA). Horseradish peroxidase-conjugated secondary antibodies were acquired from GeneTex, Inc. (Irvine, CA, USA). Isotroy was purchased from Troikaa Pharmaceuticals, Ltd. (Ahmedabad, GJ, India). All other chemicals and reagents were purchased from Sigma-Aldrich (St. Louis, MO, USA).

### 2.2. Plant Materials

Paeoniae Radix Alba (PR) was supplied by Bonchowon (Yeongcheon-si, Gyeongsangnam-do, Korea). PR (300 g) was extracted with 10 times the amount of water and boiled at 100°C for 2 h with distilled water (D.W.). After filtration, the water extracts were evaporated using a rotary evaporator at 50°C, and the solvent was evaporated *in vacuo* to give an extract with a yield of 9.3%. Then, the powder was kept at −80°C until animal experimentation.

### 2.3. Analysis of Paeoniflorin

**Figure 1:**
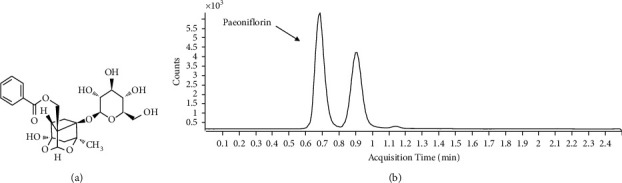
Analysis of paeoniflorin in the extract of PR. (a) Chemical structure of paeoniflorin. (b) The chromatogram of the extract of PR. PR: Paeoniae Radix Alba.

Quantitative analysis was performed using Agilent 1290 Infinity II high-performance liquid chromatograph was equipped with Agilent 6470 triple-quadrupole mass spectrometer coupled with an Agilent Jet Stream source in electrospray ionization (ESI) mode (Agilent Technologies, CA, USA). The water extract of Paeoniae Radix Alba (1 mg) was dissolved in 1 mL of 100% ACN. 100 *μ*L of the sample was mixed with an equal volume of internal standard (1 ng/mL Gliclazide in acetonitrile). Paeoniflorin separation solution (5 *μ*L) was injected onto an Agilent Zorbax Eclipse Plus C18 column (2.1 mm × 50 mm id, 1.8 *μ*m) and separated. The column and autosampler tray was kept at 40°C and 4°C, respectively. The mobile phase consisted of HPLC-grade water with 10 mM ammonium formate in distilled water as solvent A and acetonitrile as solvent B (A : B = 20 : 80 isocratic) with isocratic elution at a flow rate of 0.2 mL/min. The total run time was 2.5 min per sample. Quantification was performed with MS/MS detection set in the positive in multiple reaction monitoring modes for the analyte and IS. The amount of paeoniflorin (including 100 ng/mL) is analyzed to be 4.21 ± 0.40 ng/mL (Figure 1).

### 2.4. Experimental Animals and Induction of Acute Liver Injury

All animal experiments protocols were performed according to “The Guidelines for Animal Experiment” approved by the Ethics Committee of Daegu Haany University (Approval no. DHU2020-083). The 6-week-old male Sprague-Dawley rats (180–200 g) were purchased from DBL (Eumseong, Korea) and used for experiments after being adapted to the environment for 1 week. Environmental conditions were set to 12 h light/dark cycle, controlled humidity (50% ± 5%), and temperature (22 ± 2°C). After 1 week of adaptation, a total of thirty-five rats were randomly divided into 5 groups as follows (*n* = 7); we followed the methods of Shin et al. [18]. In addition, the concentrations of PR and silymarin were administered with reference to the doses used in the previously published acute liver injury paper [19].

1. Normal group, received D.W. only
2. Control group, TAA-induced ALI treated with D.W.
3. Silymarin group, TAA-induced ALI treated with silymarin (100 mg/kg)
4. PR100 group, TAA-induced ALI treated with PR (100 mg/kg)
5. PR200 group, TAA-induced ALI treated with PR (200 mg/kg)

All rats were weighed once a day at a certain time. Silymarin and PR were administered orally 90 min prior to TAA treatment. TAA (200 mg/kg dissolved in D.W.) was intraperitoneally administered to all groups except for the normal group every day for 3 d with drug treatment. No deaths were found in the TAA-injected group until the day of the autopsy. At the end of the experiment, rats were anesthetized by Isotroy inhalation anesthesia (induction, 4% isoflurane; maintenance, 2% isoflurane) for 5–7 min. Subsequently, blood was drawn from the abdominal vein and centrifuged at 4,800 × g for 20 min at 4°C. Next, the rats were euthanized by bleeding from the abdominal vein, and the death of the rats was confirmed through the absence of reflexes. The liver tissues were immediately stored at −80°C.

### 2.5. Measurement of Aspartate Aminotransferase (AST) and Alanine Aminotransferase (ALT) Levels

Hepatic functional parameters, such as AST and ALT, were measured using specific assay kits and a microplate fluorescence reader (Transaminase CII-Test; Wako Pure Chemical Industries, Ltd., Osaka, Japan).

### 2.6. Measurement of Ammonia Levels

Serum was collected from each group as above. Ammonia levels were measured by enzyme-linked immunosorbent assay (ELISA) kits (Abcam, Cambridge, UK) according to the manufacturer's instructions.

### 2.7. Hematoxylin and Eosin Staining of Liver Tissue

Microscopic examination was performed to evaluate the separated liver tissue. The separated liver tissue was fixed through a 10% neutral-buffered formalin and embedded in paraffin. It was cut into 2 *μ*m sections and stained using hematoxylin and eosin for microscopic evaluation. The stained slides were observed under an optical microscope and then analyzed using the i-Solution Lite software program (InnerView Co., Korea). Also, quantitative analysis (%) for inflamed cells infiltrations on H&E stains was measured on randomly selected sections from at least three fields of each sample (at x200 magnification) using image analysis with the National Institutes of Health image program (ImageJ 1.52) following the user's guide (https://imagej.net/docs/guide).

### 2.8. Western Blotting

For cytosol samples, liver tissues were lysed with buffer A consisting of 0.1 mM EDTA, 10 mM HEPES (pH 7.8), 0.1 mM phenyl methyl sulfonyl fluoride, 10 mM KCl, 1 mM DTT, 2 mM MgCl_2_, and 1,250 *μ*L protease inhibitor solution (Wako). The homogenates were incubated at 4°C for 20 min and were mixed with 10% NP-40. After centrifugation (12,000 rpm at 4°C for 2 min) using an Eppendorf 5415R (Hamburg, Germany), the supernatant was collected as the cytosol sample. After that, the lysates were suspended with 20 mL ice-cold lysis buffer C consisting of 50 mM HEPES (pH 7.8), 50 mM KCl, 300 mM NaCl, 1 mM DTT, 0.1 mM EDTA, 1% (v/v) glycerol, 0.1 mM phenyl methyl sulfonyl fluoride, and 100 *μ*L protease inhibitor solution and incubated at 4°C for 30 min. Samples were centrifuged at 12,000 rpm at 4°C for 10 min, and the supernatant collected was the nuclear sample. Both cytosol and nuclear samples were stored at −80°C before use. Samples containing 10 *μ*g of protein were electrophoresed through 8–15% SDS-PAGE and transferred to a nitrocellulose membrane. Each membrane was blocked with 5% (w/v) skim milk solution for 1 h and visualized using ECL reagents. The bands were detected by Sensi-Q 2000 ChemiDoc (Lugen Sci Co., Ltd., Gyeonggi-do, Korea). We followed the methods of Shin et al. [20].

### 2.9. Statistical Analysis

The data were expressed as the mean ± standard error of the mean (SEM). Statistical analysis was analyzed using one-way analysis of variance (ANOVA) test followed by least-significant differences (LSD) test. All analyses were performed using SPSS version 26.0 software (SPSS Inc., Chicago, IL, USA). Statistical significance levels for each group were shown at ^#,*∗*^, *p* < 0.05, ^##,*∗∗*^, *p* < 0.01, and ^###,*∗∗∗*^, *p* < 0.001.

## 3. Results

### 3.1. PR Improved Body Weight Gain, Liver Weight, Relative Liver Weight, and Liver Function

As shown in Table 1, we first measured the effects of silymarin and PR on body weight gain and relative liver weight. TAA treatment significantly decreased body weight gain (*p* < 0.001) and significantly elevated relative liver weight ratio compared with that of the normal group (*p* < 0.001). Silymarin and PR treatment improved a relative liver weight ratio without a significant difference. To evaluate the effect of silymarin and PR on liver dysfunction, serum liver function biomarkers were measured (Table 2). TAA treatment led to significant elevations of both AST and ALT. The AST and ALT levels in the TAA control group significantly increased approximately 2.2-fold and 9.8-fold, respectively, whereas silymarin and PR administration significantly decreased these levels. Moreover, silymarin and PR treatment decreased serum ammonia levels (*p* < 0.001) (control: 19.18 ± 0.74 nmol/*μ*L; silymarin: 14.73 ± 1.03 nmol/*μ*L; PR100: 12.68 ± 0.46 nmol/*μ*L; and PR200: 11.58 ± 0.73 nmol/*μ*L).

### 3.2. PR Activated p-LKB1/AMPK/Sirt1 in ALI Rat Liver Tissue

**Figure 2:**
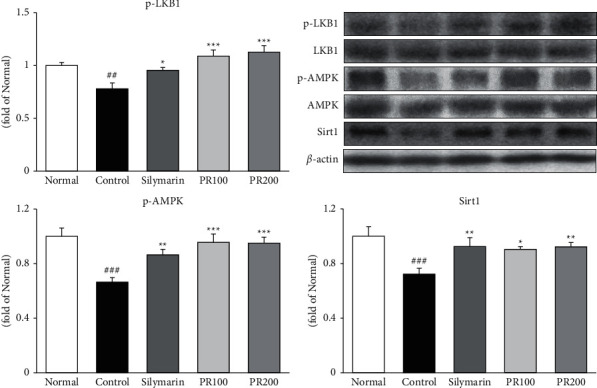
Paeoniae Radix Alba activated p-LKB1/AMP-activated protein kinase/Sirtuin 1. Paeoniae Radix Alba significantly elevated the protein expression of p-LKB1, p-AMP-activated protein kinase, and Sirtuin 1. Data are mean ± standard error of the mean (*n* = 7). ^#^*p* < 0.05, ^##^*p* < 0.01, and ^###^*p* < 0.001 versus the normal group and ^∗^*p* < 0.05, ^∗∗^*p* < 0.01, and ^∗∗∗^*p* < 0.001 versus the thioacetamide control group. AMPK: AMP-activated protein kinase; normal: received distilled water only; control: thioacetamide-induced acute liver injury treated with distilled water; silymarin: thioacetamide-induced acute liver injury treated with silymarin (100 mg/kg); PR100: thioacetamide-induced acute liver injury treated with Paeoniae Radix Alba (100 mg/kg); and PR200: thioacetamide-induced acute liver injury treated with Paeoniae Radix Alba (200 mg/kg).

Next, we investigated whether PR activates LKB1/AMPK/Sirt1 (Figure 2). Compared with the TAA control group, silymarin and PR treatment significantly increased the levels of p-LKB1, p-AMPK, and Sirt1 (silymarin, 21%, *p* < 0.05; 28%, *p* < 0.01; and 29%, *p* < 0.01; PR200, 43%, *p* < 0.001; 42%, *p* < 0.001; and 28%, *p* < 0.01, resp.). Silymarin exhibited similar efficacies of PR200 as compared with the control group in Sirt1 activation.

### 3.3. PR Suppressed the Release of NF-*κ*Bp65 and Proinflammatory Proteins in ALI Rat Liver Tissue

**Figure 3:**
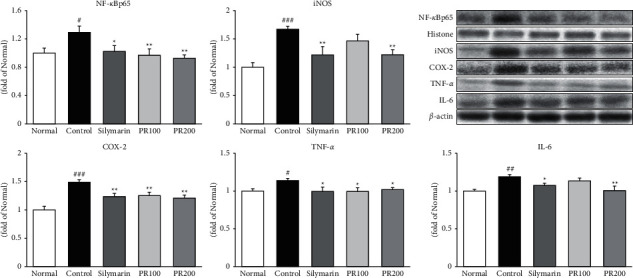
Paeoniae radix alba suppressed the release of NF-*κ*Bp65 and proinflammatory proteins. The higher dose of Paeoniae Radix Alba (200 mg/kg) significantly reduced the protein expression of NF-*κ*Bp65, iNOS, COX-2, TNF-*α*, and IL-6. Data are expressed as the mean ± standard error of the mean (*n* = 7). ^#^*p* < 0.05, ^##^*p* < 0.01, and ^###^*p* < 0.001 versus the normal group and ^∗^*p* < 0.05, ^∗∗^*p* < 0.01 versus the thioacetamide control group. Normal: received distilled water only; control: thioacetamide-induced acute liver injury treated with distilled water; silymarin: thioacetamide-induced acute liver injury treated with silymarin (100 mg/kg); PR100: thioacetamide-induced acute liver injury treated with Paeoniae Radix Alba (100 mg/kg); and PR200: thioacetamide-induced acute liver injury treated with Paeoniae Radix Alba (200 mg/kg).

TAA injection upregulated the protein levels of NF-*κ*Bp65, COX-2, iNOS, TNF-*α*, and IL-6 by 1.49-, 1.67-, 1.14-, and 1.19-fold compared with the normal group. Moreover, silymarin and PR administration significantly suppressed proinflammatory targets including NF-*κ*Bp65, COX-2, iNOS, TNF-*α*, and IL-6 (Figure 3). Herein, the ameliorative effects of the higher dose of PR (200 mg/kg) were superior to those of the lower PR dose (100 mg/kg). Compared to the pharmacological properties between silymarin and PR200, there was a tendency that GF 200 seemed better in NF-*κ*Bp65 and IL-6 levels than silymarin, but other factors, such as iNOS, COX-2, and TNF-*α*, have shown similar effects.

### 3.4. PR Alleviated the Levels of MCP-1, ICAM-1, and VCAM-1 in ALI Rat Liver Tissue

**Figure 4:**
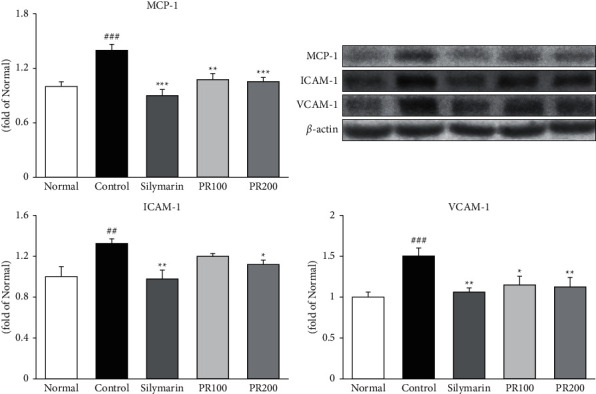
Paeoniae Radix Alba alleviated the levels of MCP-1, ICAM-1, and VCAM-1. The higher dose of Paeoniae Radix Alba (200 mg/kg) significantly reduced the protein expression of MCP-1, ICAM-1, and VCAM-1. Data are expressed as the mean ± standard error of the mean (*n* = 7). ^##^*p* < 0.01 and ^###^*p* < 0.001 versus the normal group and ^∗^*p* < 0.05, ^∗∗^*p* < 0.01, and ^∗∗∗^*p* < 0.001 versus the thioacetamide control group. Normal: received distilled water only; control: thioacetamide-induced acute liver injury treated with distilled water; silymarin: thioacetamide-induced acute liver injury treated with silymarin (100 mg/kg); PR100: thioacetamide-induced acute liver injury treated with Paeoniae Radix Alba (100 mg/kg); and PR200: thioacetamide-induced acute liver injury treated with Paeoniae Radix Alba (200 mg/kg).

The levels of MCP-1, ICAM-1, and VCAM-1 were evaluated by western blotting (Figure 4). The expression levels of MCP-1, ICAM-1, and VCAM-1 in the TAA control group were significantly higher than those in the normal group and increased by 1.40-, 1.32-, and 1.50-fold, respectively. In the silymarin and PR200 groups, the levels of MCP-1, ICAM-1, and VCAM-1 decreased significantly (silymarin: 35%, *p* < 0.001; 26% *p* < 0.01; and 29%, *p* < 0.01; PR200: 25.0%, *p* < 0.001; 15.2%, *p* < 0.05; and 16.4%, *p* < 0.01, resp.).

### 3.5. PR Abrogated Apoptosis in ALI Rat Liver Tissue

**Figure 5:**
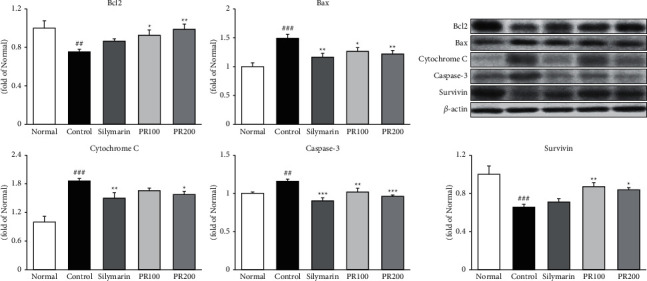
Paeoniae Radix Alba abrogated cell apoptosis. Expression of Bax, cytochrome C, and caspase-3 was significantly decreased after the administration of the higher dose of Paeoniae Radix Alba (200 mg/kg), whereas Bcl2 and survivin expression significantly increased. Data are expressed as the mean ± standard error of the mean (*n* = 7). ^###^*p* < 0.001 versus the normal group and ^∗^*p* < 0.05, ^∗∗^*p* < 0.01, and ^∗∗∗^*p* < 0.001 versus the thioacetamide control group. Normal: received distilled water only; control: thioacetamide-induced acute liver injury treated with distilled water; silymarin: thioacetamide-induced acute liver injury treated with silymarin (100 mg/kg); PR100: thioacetamide-induced acute liver injury treated with Paeoniae Radix Alba (100 mg/kg); and PR200: thioacetamide-induced acute liver injury treated with Paeoniae Radix Alba (200 mg/kg).

TAA induced apoptosis in liver tissues, as indicated by increases in Bax, cytochrome C, and cleaved caspase-3 activity (*p* < 0.001, *p* < 0.001, and *p* < 0.01, resp.) (Figure 5). In contrast, silymarin and the higher dose of PR (200 mg/kg) significantly downregulated the expression of these proteins (silymarin: *p* < 0.01, *p* < 0.01, and *p* < 0.01; PR200: *p* < 0.01, *p* < 0.05, and *p* < 0.001, resp.). PR effectively enhanced the expression of antiapoptotic proteins, such as Bcl2 and survivin (PR200: *p* < 0.01 and *p* < 0.05, resp.). These results indicated that PR inhibited hepatocyte apoptosis by suppressing the increased levels of Bax, cytochrome C, and cleaved caspase-3 and upregulating the levels of Bcl2 and survivin.

### 3.6. PR Improved Histological Alterations in ALI Rat Liver Tissue

**Figure 6:**
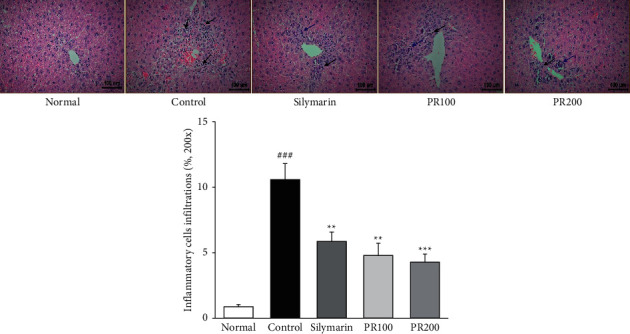
Paeoniae Radix Alba alleviated histological alterations in the liver. Representative hematoxylin and eosin staining of thioacetamide-induced acute liver injury. Injury was notably ameliorated by the administration of Paeoniae Radix Alba. Scale bar = 100 *μ*m (magnification, ×200). Blue arrows indicate inflammatory cell infiltrations on H&E sections. Black arrows indicate apoptotic body. Histological analysis was measured using ImageJ (NIH). Data are expressed as the mean ± standard error of the mean (*n* = 7). ^###^*p* < 0.001 versus the normal group and ^∗∗^*p* < 0.01 and ^∗∗∗^*p* < 0.001 versus the thioacetamide control group. Normal: received distilled water only; control: thioacetamide-induced acute liver injury treated with distilled water; silymarin: thioacetamide-induced acute liver injury treated with silymarin (100 mg/kg); PR100: thioacetamide-induced acute liver injury treated with Paeoniae Radix Alba (100 mg/kg); and PR200: thioacetamide-induced acute liver injury treated with Paeoniae Radix Alba (200 mg/kg).

Histological changes in the liver tissues of the rats were examined using hematoxylin and eosin staining. Representative images are shown in Figure 6. Treatment with TAA led to the elevation of liver volume and showed rough and granular apparatus of the liver surface. Silymarin and PR treatment relieved these liver lesions. The livers in the normal group did not present any structural changes. However, the livers in the TAA control group showed inflammatory cells infiltration (blue arrows) and necrosis of hepatocytes. Apoptotic cells show abnormal sizes, nuclear pyknosis, and karyorrhexis (black arrows). Herein, silymarin and PR treatment effectively recovered the injured livers (silymarin, *p* < 0.01; PR100, *p* < 0.001; and PR200, *p* < 0.001).

## 4. Discussion

ALI is defined as liver dysfunction and persistent liver injury that can lead to liver fibrosis and is a fundamental initiating factor in the outbreak of liver cirrhosis or hepatocellular carcinoma.

Although the etiological factor of ALI is not clear, it is known that it is caused by various causes, such as drug abuse, food additives, viral infection, alcohol, and radioactive damage [21]. It is known that ALI caused by these causes inflammation, hepatocellular necrosis, and oxidative stress [22]. However, there are no therapeutics for curative without side effects to cure liver injury globally till nowadays.

PR (white peony root) has been used in various prescriptions of traditional Chinese medicine. It is usually indicated for viral infections, menstrual disorders, and painful conditions [6]. In addition, PR is known for its functions of nourishing blood, preventing perspiration, astringing Yin, and eradicating liver wind caused by extreme Qi/Blood and Yin/Yang imbalance [23]. A recent study suggested that the bioactive component (polysaccharides) obtained from PR inhibited hepatic infiltration of inflammatory cells and overexpression of proinflammatory cytokines [9]. Moreover, paeoniflorin, a glucoside of PR, has been used to treat hepatic inflammatory disease including liver ischemia/reperfusion injury [24]. These studies indicate that PR possesses potent pharmacological activity associated with liver diseases. Therefore, using PR, a natural plant, we studied the mechanism of action of PR on liver damage that was different from studies reported so far, and we aimed to study the development of a natural therapeutic agent for liver damage based on PR.

A TAA-induced ALI model is a typical animal model that is commonly used to study the liver and its underlying mechanisms. The accumulating report indicates that TAA is a toxic substance that causes liver disease through abnormal detoxification that can cause liver tissue damage [25]. The highly toxic reactive intermediate (thioacetamide S-dioxide) of TAA damages the integrity and stability of biological membranes, which increases the permeability resulting in the outflow of enzymes, such as AST and ALT. Therefore, the determination of AST and ALT activities is a substantial index for judging liver injury [26]. Moreover, ammonia is the waste produced by nitrogen metabolism that is usually transported through the portal circulation to periportal hepatocytes. Hyperammonemia is a life-threatening metabolic syndrome characterized by elevated levels of toxic ammonia and can lead to a serious neurologic emergency that can induce liver cirrhosis, intracranial pressure crises, cerebral edema, hepatic encephalopathy, and seizures [27]. Thus, we addressed the effects of PR on TAA-induced liver injury. As expected, the serum AST, ALT, and ammonia levels of the PR-treated group were significantly lower than those of the TAA control group, indicating that it was more effective than the positive control group, silymarin. Therefore, this suggests that PR may exert a protective effect on TAA-induced ALI.

Sirt1 deacetylates LKB1 kinase, which is the upstream activator of AMPK and provides positive feedback between AMPK and Sirt1. LKB1 directly activates AMPK and has an established function in the control of OS and cell metabolism [28]. AMPK is involved in energy expenditure in specialized metabolic tissue including the liver by improving Sirt1 activity by increasing cellular NAD^+^ level [29]. Sirt1 has been reported to modulate various activities through controlling oxidative stress response, inflammation, apoptosis, metabolism, and mitochondrial function [30]. For that reason, researchers have recently focused on the discovery of Sirt1 modulators that can lead to Sirt1 activity. The results of this present study indicated that the activities of both AMPK and Sirt1 were noticeably reduced in TAA-induced ALI rats. However, silymarin and PR treatment significantly increased the activities of both AMPK and Sirt1 via the deacetylation of LKB1. Apart from oxidative energy production, Sirt1 also inhibits NF-*κ*B inflammatory signaling [31]. The activation of NF-*κ*B is involved in the overproduction of inflammatory proteins including COX-2, iNOS, TNF-*α*, and IL-6 and chemokines including MCP-1. Meanwhile, cell adhesion molecules, such as ICAM-1 and VCAM-1, are elevated by macrophage activation [32, 33]. Namely, NF-*κ*B acts as a common transcriptional regulator on the inflammatory response and exerts fundamental functions, such as cell survival and immune responses, in the liver. Notably, the expression of MCP-1, ICAM-1, and VCAM-1 was rapidly increased through stimulation with the toxic factor TAA. The high level of ICAM-1 improves the adhesion of monocytes to endothelial cells, VCAM-1 can promote monocyte adhesion to endothelial cells, and MCP-l can activate monocytes by binding with receptors [34]. In this study, we evaluated the inhibitory effect of PR on NF-*κ*B activation in TAA-induced ALI rats. The increased NF-*κ*B levels by TAA induction was remarkably suppressed in PR-treated groups. The silymarin and higher dose of PR (200 mg/kg) led to significant inhibition of proinflammatory proteins, chemokines, and cell adhesion molecules.

Excessive exposure to reactive oxygen species under inflammatory conditions increases the death of liver cells via the two standard mechanisms of apoptosis or necrosis. Apoptosis is the first response step of liver cells to diverse injury factors, and necrosis tends to occur following apoptosis. Accordingly, the apoptosis process takes charge of a key role in the formation of necrosis [35]. Many genes are involved in the apoptosis process, and of these, the activation of Bax has promoted mitochondrial membrane permeability and induces apoptosis and antagonizes the inhibitory action of Bcl-2 on apoptosis [36]. In addition, Bax forms a Bcl-2-Bax heterodimer to inhibit Bcl-2 function or induce apoptosis independently without Bcl-2 [37, 38]. Activation of caspase-3 also activates apoptosis by inducing leakage of cytochrome C, which triggers intrinsic apoptosis [39], whereas survivin in the cytoplasm inhibits caspase-3 activity or binds to the proapoptotic protein SMAC/DIABLO to inhibit apoptosis. Thus, the balance between these proteins is an important part of hepatocyte apoptosis [40–42]. Thus, further study on the mechanism of apoptosis is important to reveal the nature of ALI. Thus, we postulated that TAA injection is responsible for causing apoptotic signals through the regulation of apoptosis-related proteins including Bax, Bcl2, cytochrome C, caspase-3, and survivin. Treatment with the silymarin and higher dose of PR (200 mg/kg) showed a considerable decrease of proapoptotic genes, Bax, cytochrome C, and caspase-3. The higher dose of PR exhibited a significant increase of antiapoptotic genes, Bcl2, and survivin.

## 5. Conclusions

**Figure 7:**
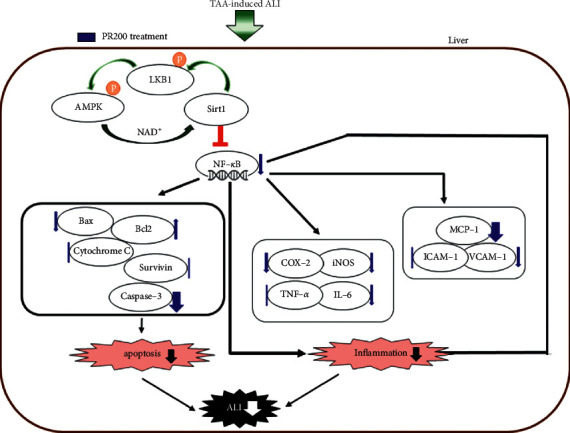
Possible mechanism of Paeoniae Radix Alba in thioacetamide-induced acute liver injury. TAA: thioacetamide; ALI: acute liver injury; PR200: thioacetamide-induced acute liver injury treated with Paeoniae Radix Alba (200 mg/kg); AMPK: AMP-activated protein kinase; and Sirt1: sirtuin 1.

Taken together, the hepatoprotective effect of PR was revealed through the inhibition of TAA-induced inflammation via the AMPK/Sirt1/NF-*κ*B pathway, as shown in Figure 7. This study demonstrated that PR could be developed as an effective therapeutic option for liver protection. Nevertheless, further research on PR is needed to increase both the safety and the therapeutic potential.

## Figures and Tables

**Table 1:** Change of body weight, liver weight, and a relative liver weight.

Group	Liver weight (g)	Body weight (g)	LW/BW ratio (%)
Normal	8.3 ± 0.11	238.9 ± 2.08	3.5 ± 0.05
Control	10.1 ± 0.21^###^	202.9 ± 3.48^###^	5.0 ± 0.10^###^
Silymarin	9.8 ± 0.13^*∗∗*^	202.5 ± 2.53^*∗∗*^	4.8 ± 0.06^*∗∗∗*^
PR100	9.8 ± 0.17^*∗∗*^	200.2 ± 3.06^*∗∗*^	4.9 ± 0.08^*∗∗∗*^
PR200	9.5 ± 0.24^*∗∗∗*^	197.4 ± 4.09^*∗∗*^	4.8 ± 0.11^*∗∗∗*^

Data are represented as mean ± standard error of the mean (*n* = 7). ^###^*p* < 0.001 versus the normal group and ^*∗∗*^*p* < 0.01 and ^*∗∗∗*^*p* < 0.001 versus the thioacetamide control group. LW: liver weight; BW: body weight; normal: received distilled water only; control: thioacetamide-induced acute liver injury treated with distilled water; (3) silymarin: thioacetamide-induced acute liver injury treated with silymarin (100 mg/kg); PR100: thioacetamide-induced acute liver injury treated with Paeoniae Radix Alba (100 mg/kg); and PR200: thioacetamide-induced acute liver injury treated with Paeoniae Radix Alba (200 mg/kg).

**Table 2:** Levels of AST, ALT, and ammonia in serum.

Group	AST (IU/L)	ALT (IU/L)	Ammonia (nmoL/*μ*L)
Normal	26.67 ± 1.39	2.74 ± 0.54	11.62 ± 0.62
Control	58.75 ± 2.75^###^	26.48 ± 2.37^###^	19.18 ± 0.74^###^
Silymarin	50.06 ± 1.98^*∗∗*^	17.07 ± 2.13^*∗∗*^	14.73 ± 1.03^*∗∗∗*^
PR100	49.01 ± 2.34^*∗∗*^	14.99 ± 3.65^*∗∗*^	12.68 ± 0.46^*∗∗∗*^
PR200	44.73 ± 2.09^*∗∗∗*^	14.50 ± 3.54^*∗∗*^	11.58 ± 0.73^*∗∗∗*^

The higher dose of Paeoniae Radix Alba (200 mg/kg) significantly reduced the levels of AST, ALT, and ammonia. Data are mean ± standard error of the mean (*n* = 7). ^###^*p* < 0.001 versus the normal group and ^*∗∗*^*p* < 0.01 and ^*∗∗∗*^*p* < 0.001 versus the thioacetamide control group. Normal: received distilled water only; control: thioacetamide-induced acute liver injury treated with distilled water; silymarin: thioacetamide-induced acute liver injury treated with silymarin (100 mg/kg); PR100: thioacetamide-induced acute liver injury treated with Paeoniae Radix Alba (100 mg/kg); and PR200: thioacetamide-induced acute liver injury treated with Paeoniae Radix Alba (200 mg/kg).

## Data Availability

The data sets used and analyzed during this study are available from the corresponding author upon reasonable request.
